# Selective NADH communication from α-ketoglutarate dehydrogenase to mitochondrial transhydrogenase prevents reactive oxygen species formation under reducing conditions in the heart

**DOI:** 10.1007/s00395-020-0815-1

**Published:** 2020-08-03

**Authors:** Michael Wagner, Edoardo Bertero, Alexander Nickel, Michael Kohlhaas, Gary E. Gibson, Ward Heggermont, Stephane Heymans, Christoph Maack

**Affiliations:** 1 Clinic III for Internal Medicine, University Clinic Homburg, 66421 Homburg, Germany; 2 Present Address: Department of Translational Research, Comprehensive Heart Failure Center (CHFC), University Clinic Würzburg, Am Schwarzenberg 15, Haus A15, 97078 Würzburg, Germany; 3 Brain and Mind Research Institute, Weill Cornell Medicine, Burke Neurological Institute, 785 Mamaroneck Avenue, White Plains, NY 10605 USA; 4 Cardiovascular Research Center, OLV Hospital Aalst, Moorselbaan 164, 9300 Aalst, Belgium; 5 Department of Cardiology, CARIM School for Cardiovascular Diseases Faculty of Health, Medicine and Life Sciences, Maastricht University, Maastricht, The Netherlands; 6 Department of Cardiovascular Sciences, Centre for Molecular and Vascular Biology, KU Leuven, Belgium; 7 The Netherlands Heart Institute, Nl-HI, Utrecht, The Netherlands

**Keywords:** Mitochondria, α-Ketoglutarate dehydrogenase, Reactive oxygen species, Nicotinamide nucleotide transhydrogenase

## Abstract

In heart failure, a functional block of complex I of the respiratory chain provokes superoxide generation, which is transformed to H_2_O_2_ by dismutation. The Krebs cycle produces NADH, which delivers electrons to complex I, and NADPH for H_2_O_2_ elimination via isocitrate dehydrogenase and nicotinamide nucleotide transhydrogenase (NNT). At high NADH levels, α-ketoglutarate dehydrogenase (α-KGDH) is a major source of superoxide in *skeletal* muscle mitochondria with low NNT activity. Here, we analyzed how α-KGDH and NNT control H_2_O_2_ emission in *cardiac* mitochondria. In cardiac mitochondria from NNT-competent BL/6N mice, H_2_O_2_ emission is equally low with pyruvate/malate (P/M) or α-ketoglutarate (α-KG) as substrates. Complex I inhibition with rotenone increases H_2_O_2_ emission from P/M, but not α-KG respiring mitochondria, which is potentiated by depleting H_2_O_2_-eliminating capacity. Conversely, in NNT-deficient BL/6J mitochondria, H_2_O_2_ emission is higher with α-KG than with P/M as substrate, and further potentiated by complex I blockade. Prior depletion of H_2_O_2_-eliminating capacity increases H_2_O_2_ emission from P/M, but not α-KG respiring mitochondria. In cardiac myocytes, downregulation of α-KGDH activity impaired dynamic mitochondrial redox adaptation during workload transitions, without increasing H_2_O_2_ emission. In conclusion, NADH from α-KGDH selectively shuttles to NNT for NADPH formation rather than to complex I of the respiratory chain for ATP production. Therefore, α-KGDH plays a key role for H_2_O_2_ elimination, but is not a relevant source of superoxide in heart. In heart failure, α-KGDH/NNT-dependent NADPH formation ameliorates oxidative stress imposed by complex I blockade. Downregulation of α-KGDH may, therefore, predispose to oxidative stress in heart failure.

**Electronic supplementary material:**

The online version of this article (10.1007/s00395-020-0815-1) contains supplementary material, which is available to authorized users.

## Introduction

Mitochondria are the major source of cellular adenosine triphosphate (ATP), but also reactive oxygen species (ROS). Chronic heart failure is associated with derangements at multiple levels in the processes of substrate utilization, mitochondrial oxidative metabolism, and shuttling of ATP from mitochondria to the cytosol [2], and these abnormalities lead to energetic deficit [25, 26] and oxidative stress [21]. In heart failure, ROS production is increased in mitochondria [11], but also from other sources, such as NADPH oxidase [9, 21] and xanthine oxidase [5]. Within mitochondria, canonical sites for ROS formation are complexes I and III of the electron transport chain (ETC) [1], and a functional block of complex I provokes elevated ROS production in heart failure [11].

The overflow of ROS from mitochondria in heart failure is not solely explained by the increase in ROS formation, but is also accounted for by the inability of mitochondria to efficiently eliminate ROS [27]. Conversion of hydrogen peroxide (H_2_O_2_) to water is catalyzed by glutathione peroxidase and peroxiredoxin, which are in turn regenerated by a cascade of redox reactions requiring the reduced form of nicotinamide adenine dinucleotide phosphate (NADPH) as an electron donor. Furthermore, catalase might contribute to H_2_O_2_ elimination in cardiac mitochondria [31], and NADPH is required to protect catalase against inactivation by its substrate [12, 13]. In the failing heart, the dynamic regulation of the mitochondrial antioxidative capacity is hampered by maladaptive changes of excitation–contraction (EC) coupling [3]. Specifically, decreased calcium (Ca^2+^) release from the sarcoplasmic reticulum [16] and elevated cytosolic sodium (Na^+^) concentrations [15, 19] in failing cardiac myocytes decrease Ca^2+^ uptake and accelerate Ca^2+^ efflux from the mitochondrial matrix, respectively. Consequently, the Ca^2+^-dependent stimulation of the Krebs cycle dehydrogenases is hindered, leading to a decrease in the NAD(P)H/NAD(P)^+^ ratio. Oxidation of mitochondrial pyridine nucleotides limits the supply of electrons to the ETC [22] and to the antioxidant systems required for H_2_O_2_ elimination [15]. Thus, disrupted intracellular Ca^2+^ handling in heart failure limits regeneration of antioxidative capacity, resulting in ROS emission from mitochondria [15, 17].

The mitochondrial nicotinamide nucleotide transhydrogenase (NNT) represents an essential interface between the NADPH and NADH pools in the heart. Under physiological conditions, the NNT harnesses the protonmotive force (Δμ_H_) to transfer hydride ion equivalents (H^−^) from NADH to NADPH, thus maintaining the mitochondrial NADPH/NADP^+^ ratio several fold higher than the NADH/NAD^+^ ratio. In the heart, pathological elevations of workload reverse the direction of the NNT reaction, which then consumes NADPH to regenerate NADH, thereby depleting mitochondrial antioxidative capacity. This model is corroborated by the observation that the C57BL/6J mouse strain (BL/6J), which carries a loss-of-function mutation of *Nnt* [37], is protected from mitochondrial oxidative stress and maladaptive cardiac remodeling upon pressure overload compared with the C57BL/6N strain (BL/6N), which harbors a functional NNT [28].

In addition to the ETC complexes, numerous other sites within mitochondria might become a source of ROS under certain conditions [24]. Studies performed in isolated mitochondria indicate that the α-ketoglutarate (α-KG) dehydrogenase (α-KGDH) complex, which catalyzes the rate-limiting reaction of the Krebs cycle [6], is the major source of mitochondrial ROS under conditions of elevated NADH/NAD^+^ ratios [30, 34, 38]. The α-KGDH complex catalyzes the reaction of α-ketoglutarate (α-KG) and the cofactors thiamine pyrophosphate, NAD^+^ and coenzyme A to succinyl-CoA, CO_2_, and NADH. It is a multienzyme complex consisting of multiple copies of the three subunits α-ketoglutarate dehydrogenase (OGDH, or E1 component), dihydrolipoyl succinyltransferase (DLST, or E2 component), and dihydrolipoyl dehydrogenase (DLD, or E3 component) [36]. Experiments performed in isolated α-KGDH preparations indicate that the flavin group of the DLD (E3) component of the complex might act as electron donor for superoxide formation, thus accounting for the high rate of H_2_O_2_ emission from mitochondria respiring on α-KG [10]. Furthermore, α-KGDH is also susceptible to oxidative damage, and thus represents both a target and a source of mitochondrial ROS [35].

On the other hand, our own previous studies imply that NAD(P)H regeneration by the Krebs cycle dehydrogenases is required to maintain mitochondrial antioxidative capacity, and thereby prevent overflow of ROS from cardiac mitochondria [15, 28]. Importantly, NADH derived from α-KGDH reaction might contribute to NADPH regeneration via the NNT, but experiments pinpointing α-KGDH as a major source of ROS were performed in skeletal muscle or brain mitochondria [30, 34, 38], where NNT activity is substantially lower than in the heart [28]. Unraveling the role of α-KGDH in the redox balance of cardiac mitochondria has important implications for heart failure, since we recently observed that pathological elevations of cardiac workload decrease α-KGDH expression and activity secondary to microRNA146a-mediated downregulation of the DLST subunit [8].

Here, we analyzed NAD(P)H production and respiration of isolated cardiac mitochondria supplied with α-KG or the complex I substrates pyruvate and malate (P/M), and assessed whether NADH generated by the α-KGDH complex contributes to mitochondrial antioxidative capacity via the NNT in cardiac mitochondria. Furthermore, we investigated how impaired α-KGDH activity affects the Ca^2+^-dependent regeneration of NAD(P)H when mitochondria are integrated in their physiological context of intact cardiac myocytes. Altogether, our results indicate that NADH produced by α-KGDH is preferentially used to regenerate antioxidative NADPH via the NNT, suggesting an important antioxidant role of the α-KGDH and arguing against its role as a primary ROS source in cardiac mitochondria.

## Materials and methods

### Ethical approval and animal experiments

All animal experiments were performed in accordance to guidelines of the local animal ethics committee. C57BL/6 mice were purchased from Charles River (BL/6N, C57BL/6NCrl, strain code 027; and BL/J, C57BL/6J, JAX Mice Stock Number 000664). *Dlst*^+/−^ mice and corresponding wild-type (WT) littermates were generated by Prof. Gary E. Gibson (Weill Cornell Medical College, New York) and received through Prof. Stéphane Heymans and Dr. Ward Heggermont (KU Leuven).

**Fig. 1:**
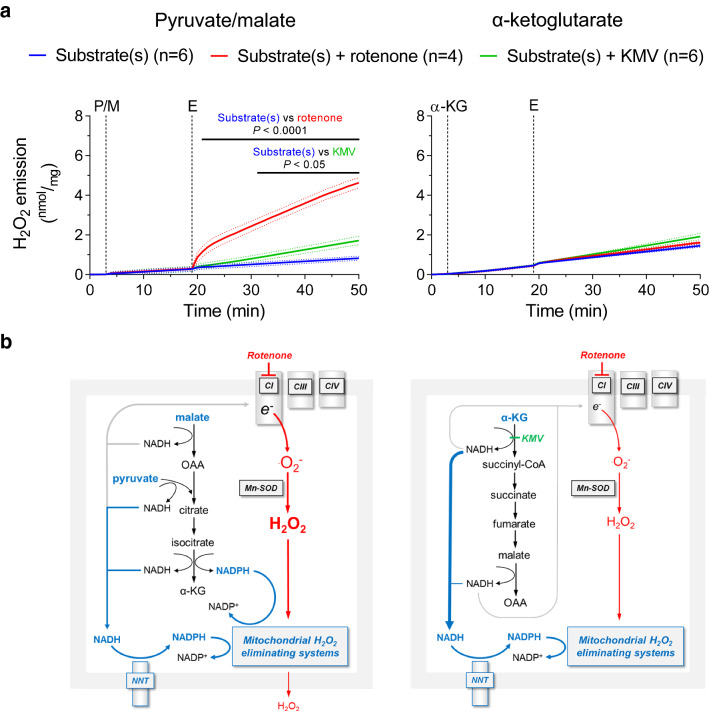
H_2_O_2_ emission from cardiac mitochondria supplied with pyruvate/malate or α-ketoglutarate. **a** H_2_O_2_ emission (by Amplex^®^ UltraRed) of isolated cardiac mitochondria harvested from BL/6N mice was measured in the presence of pyruvate and malate (P/M, 5 mM each) or α-ketoglutarate (α-KG, 5 mM) as substrates (added at time point S) and in response to addition of DMSO (blue trace), the complex I inhibitor rotenone (8 µM, red trace) or the α-ketoglutarate dehydrogenase (α-KGDH) inhibitor KMV (10 mM, green trace) at time point E (effectors). **b** The mitochondrial hydrogen peroxide (H_2_O_2_)-eliminating systems, i.e. glutathione peroxidase and peroxiredoxin, are maintained in their active form by a series of redox reactions requiring NADPH as an electron donor. In mitochondria respiring on (P/M) (left panel), NADPH regeneration is mainly mediated by the nicotinamide nucleotide transhydrogenase (NNT) and the isocitrate dehydrogenase type 2 reactions. Upon complex I inhibition with rotenone, the increase in the NADH/NAD^+^ ratio induces leakage of electrons and consequent superoxide (^.^O_2_^−^) formation at complex I of the respiratory chain. The mitochondrial superoxide dismutase (Mn-SOD) converts ^.^O_2_^−^ to H_2_O_2_, which overcomes the mitochondrial detoxifying capacity and accounts for the H_2_O_2_ emission observed in (**a**). In BL/6N mitochondria supplied with α-KG (right panel), the NADH produced via the α-KGDH reaction is preferentially used for NADPH regeneration via the NNT rather than being shuttled to complex I, where it would induce ^.^O_2_^−^ formation, thereby leading to very low levels of H_2_O_2_ emission as seen in (**a**). Results are shown as mean ± standard error of the mean; *n* = number of mice. Significance was calculated using two-way ANOVA with Bonferroni’s post-hoc test. Continuous black lines above traces indicate statistically significant differences (defined as *P* < 0.05) between experimental groups by Bonferroni’s post-hoc test

**Fig. 2:**
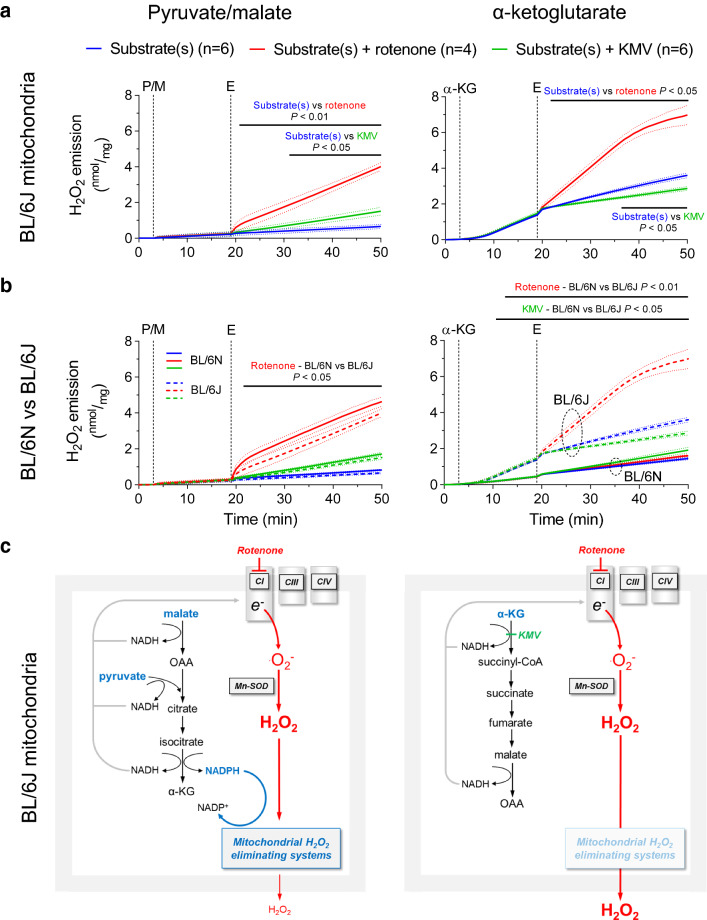
H_2_O_2_ emission from cardiac mitochondria of BL/6J mice supplied with pyruvate/malate or α-ketoglutarate. **a** H_2_O_2_ emission (by Amplex^®^ UltraRed) of isolated cardiac mitochondria harvested from mice lacking a functional NNT (BL/6J) was measured in the presence of pyruvate and malate (P/M, 5 mM each) or α-ketoglutarate (α-KG, 5 mM) as substrates (added at time point S) and in response to addition of DMSO (blue trace), the complex I inhibitor rotenone (8 µM, red trace) or the α-ketoglutarate dehydrogenase (α-KGDH) inhibitor KMV (10 mM, green trace) at time point E (effectors). **b** Direct comparison of H_2_O_2_ emission from BL/6N and BL/6J mitochondria supplied with (P/M) or (α-KG) [data from Fig. 1a and panel (a)]. **c** In mitochondria lacking a functional NNT (BL/6J) provided with P/M (left panel), the isocitrate dehydrogenase reaction is sufficient to maintain mitochondrial H_2_O_2_-eliminating capacity, leading to a rate of H_2_O_2_ emission comparable to that of BL/6N mitochondria. In contrast, the mitochondrial antioxidative capacity cannot be regenerated in BL/6J mitochondria supplied with α-KG alone (right panel), and α-KGDH-derived NADH fuels ROS production at the respiratory chain when complex I is inhibited with rotenone, leading to the highest rate of H_2_O_2_ emission observed in the four experimental conditions. Results are shown as mean ± standard error of the mean; *n* = number of mice. Significance was calculated using two-way ANOVA with Bonferroni’s post-hoc test. Continuous black lines above traces indicate statistically significant differences (defined as *P* < 0.05) between experimental groups by Bonferroni’s post-hoc test

**Fig. 3:**
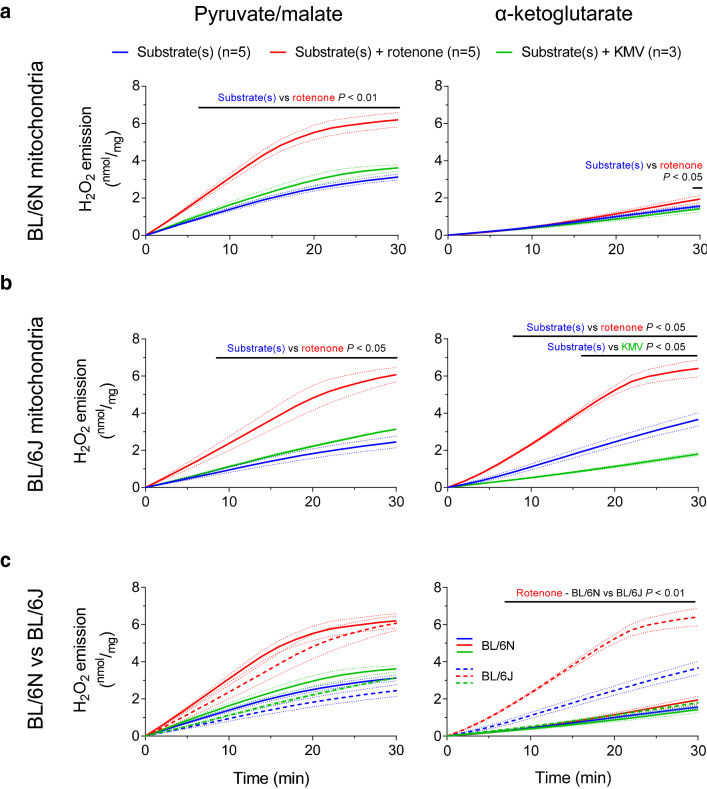
H_2_O_2_ emission from cardiac mitochondria depleted of their antioxidative capacity. H_2_O_2_ emission (by Amplex^®^ UltraRed) of isolated cardiac mitochondria harvested from BL/6N (**a**) or BL/6J (**b**) mice was measured after incubation with with 2,4-dinitrochlorobenzene, which binds covalently to glutathione. Pyruvate and malate (5 mM each) or α-ketoglutarate (5 mM) were used as substrates. H_2_O_2_ emission was measured in the presence of vehicle (black trace), substrate(s) alone (blue trace), rotenone (8 µM, red trace) or the α-ketoglutarate dehydrogenase inhibitor KMV (10 mM, green trace). The direct comparison between BL/6N and BL/6J mitochondria is shown in (**c**). Results are shown as mean ± standard error of the mean; *n* = number of mice. Significance was calculated using two-way ANOVA with Bonferroni’s post-hoc test. Continuous black lines above traces indicate statistically significant differences (defined as *P* < 0.05) between experimental groups by Bonferroni’s post-hoc test

**Fig. 4:**
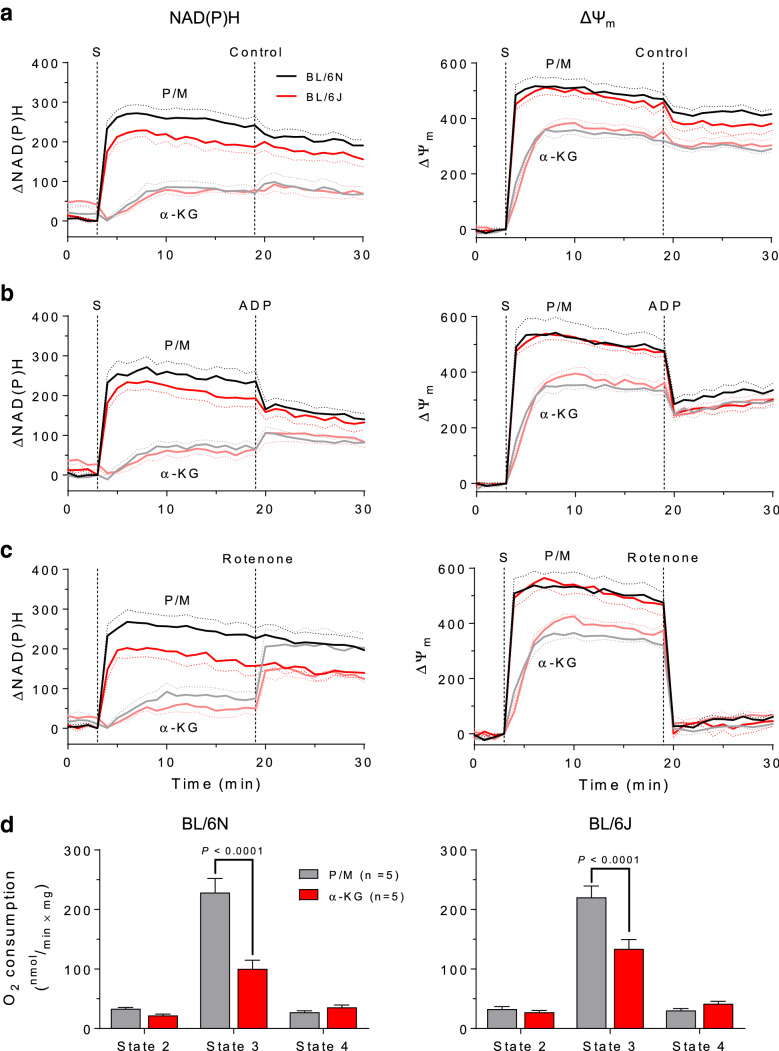
NAD(P)H, membrane potential and respiration of cardiac mitochondria supplied with pyruvate/malate or α-ketoglutarate. NAD(P)H autofluorescence (left) and mitochondrial membrane potential (ΔΨ_m,_ measured with the potentiometric dye TMRM, right) of cardiac mitochondria from BL/6N (black and gray traces) and BL/6J (red traces) mice supplied with pyruvate and malate (P/M, 5 mM each) or α-ketoglutarate (α-KG, 5 mM) as substrates (added at time point S). The effects of DMSO (as control, **a**), ADP (1 mM, **b**) and the complex I inhibitor rotenone (8 µM, **c**) on NAD(P)H autofluorescence and ΔΨ_m_ did not differ between BL/6N and BL/6J mitochondria. **d** Respiration of isolated mitochondria from BL/6N (left) and BL/6J (right) mice supplied with P/M or α-KG. O_2_ consumption was measured in the presence of substrate(s) alone (state 2), upon ADP addition (1 mM, state 3) and after inhibiting ADP phosphorylation with oligomycin (1.2 µM, state 4). Results are shown as mean ± standard error of the mean; *n* = number of mice. Significance was calculated using two-way ANOVA with Bonferroni’s post-hoc test

For cardiac myocyte isolation, mice received carprofen (12 mg kg^–1^) and heparin (10,000 IE kg^–1^) and were anaesthetized with isoflurane (5% at 0.5 L min^–1^). The heart was excised as soon as mice became insensible to pedal reflex. For mitochondrial isolation from mouse hearts, mice were sacrificed by intraperitoneal injection of ketamine hydrochloride (100 mg kg^–1^; Pfizer, Karlsruhe, Germany) and xylazine hydrochloride (10 mg kg^–1^; Bayer Healthcare, Berlin, Germany) (Fig. 1, 2, 3, 4).

### Experiments in isolated cardiac myocytes

**Fig. 5:**
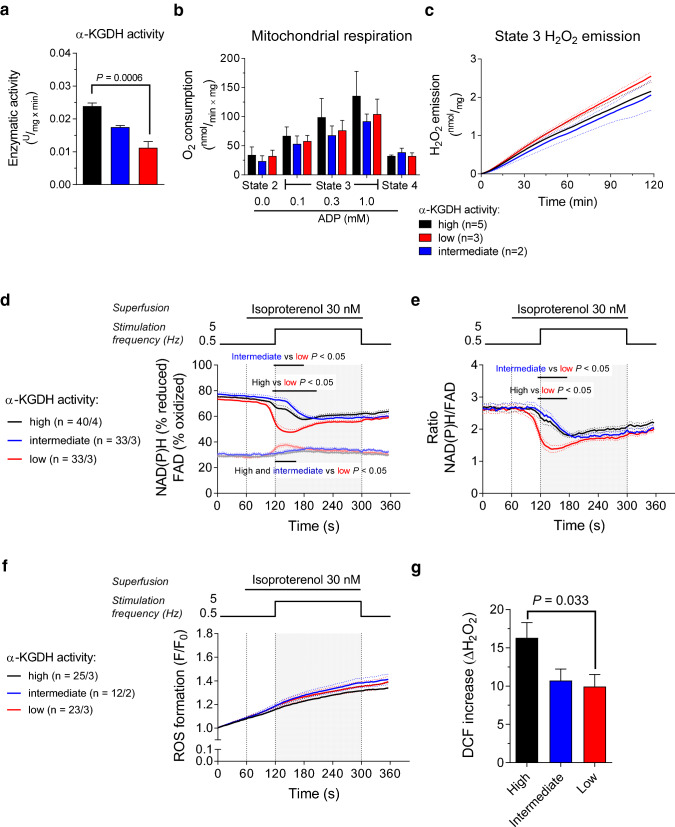
Mitochondrial respiration, redox state, and ROS production of isolated mitochondria and cardiac myocytes grouped based on α-ketoglutarate dehydrogenase activity. Enzymatic activity of α-KGDH **a** in the heart of *Dlst*^+/−^ mice and wild-type littermates divided in groups of high, intermediate and low α-KGDH activity. O_2_ consumption **b** and H_2_O_2_ emission (by Amplex^®^ UltraRed, **c** of isolated cardiac mitochondria supplied with α-ketoglutarate as substrate and grouped based on their respective α-ketoglutarate dehydrogenase activity. In (**b**), O_2_ consumption was measured in the presence of α-ketoglutarate alone (state 2), upon ADP addition (state 3) and after inhibiting ADP phosphorylation with oligomycin (1.2 µM, state 4). In (**c**), H_2_O_2_ emission was measured in the presence of saturating ADP (1 mM). NAD(P)H and FAD autofluorescence (**d**), NAD(P)H/FAD ratio (**e**), and ROS production (with the fluorescent ROS indicator DCF, **f** of isolated, field-stimulated cardiac myocytes grouped based on their α-ketoglutarate dehydrogenase (α-KGDH) activity. To simulate an increase in workload, cardiac myocytes were superfused with the β-agonist isoproterenol (30 nM) and stimulation frequency was increased from 0.5 to 5 Hz for 180 s (corresponding to the gray-shaded area). To assess H_2_O_2_-eliminating capacity, cardiac myocytes were superfused with external H_2_O_2_ (10 mM) and the increase in DCF fluorescence was measured (**g**). Results are shown as mean ± standard error of the mean; *n* = number of mice for panels (**a**) to (**c**) and number of cells/number of mice for panels (**d**) to (**f**). Significance was calculated using one-way ANOVA with Bonferroni’s post-hoc test for panels (**a**) and (**g**) and two-way ANOVA with Bonferroni’s post-hoc test for panels (**b**) to (**f**). *P*-values in panels (**a**) and (**g**) indicate statistically significant differences (defined as *P* < 0.05) by Bonferroni’s post-hoc test. In panels (**d**) and (**e**), continuous black lines above traces indicate statistically significant differences between experimental groups by Bonferroni’s post-hoc test

Most methods employed in this study were described previously [28]. For the experiments in Fig. 5d–g and **Supplementary Fig. 4**, isolated murine ventricular myocytes were field-stimulated at 0.5 Hz and perfused with Normal Tyrode’s (NT) solution containing (in mM): NaCl 130, KCl 5, MgCl_2_ 1, CaCl_2_ 1, Na-HEPES 10, glucose 10, sodium pyruvate 2 and ascorbic acid 0.3, pH 7.4. After 120 s, cardiac myocytes were perfused with NT solution containing the β-adrenergic receptor agonist isoproterenol (30 nM) and pacing frequency was increased to 5 Hz for 180 s. Finally, isoproterenol was washed out by perfusing the cells again with NT, and a 0.5 Hz pacing frequency was restored. During this experimental stress protocol, sarcomere length, NAD(P)H and FAD autofluorescence, and 5-(-6)-chloromethyl-2,7-dichlorohydrofluorescein di-acetate (CM-H_2_DCFDA, denoted as DCF in the Results) fluorescence were measured with an IonOptix setup.

### Experiments in isolated mitochondria

Mitochondria were isolated from murine hearts as previously described [28]. O_2_ consumption was measured at 37 °C with a Clarke electrode (Hansatech, Pentney, Norfolk, UK). State 2 respiration was obtained using sodium pyruvate and sodium malate or α-KG as substrates (5 mM each). Increasing concentrations of ADP (0.03, 0.1, 0.3 and 1 mM) or a single addition of a saturating concentration of ADP (1 mM) were used to induce state 3 respiration. Finally, state 4 respiration was induced by adding the F_1_/F_o_ ATP synthase (complex V) inhibitor oligomycin (1.2 µM).

Hydrogen peroxide (H_2_O_2_) emission from isolated cardiac mitochondria was determined using the H_2_O_2_-specific dye Amplex^®^ UltraRed (purchased from Thermo Fisher Scientific, Waltham, Massachusetts, USA). The reaction of Amplex^®^ UltraRed with H_2_O_2_ is catalyzed by horseradish peroxidase (HRP) and forms the fluorescent product resorufin (*λ*_Ex_ = 535 nm, *λ*_Em_ = 590 nm). The concentrations used in the assay were 10 µM Amplex^®^ UltraRed, 0.5 U mL^−1^ HRP and 100 U mL^−1^ superoxide dismutase. H_2_O_2_ emission was measured in the presence of ADP (1 mM), the complex I inhibitor rotenone (8 µM) or the α-KGDH inhibitor 3-methyl-2-oxovaleric acid (KMV, 10 mM). To deplete mitochondrial antioxidative capacity, mitochondria were pretreated with 2,4-dinitrochlorobenzene (DNCB, 25 µM; purchased from Sigma-Aldrich), which alkylates glutathione and is an irreversible inhibitor of thioredoxin reductase.

For simultaneous measurement of NAD(P)H and mitochondrial membrane potential (ΔΨ_m_), isolated cardiac mitochondria were preincubated with the potentiometric marker tetramethylrhodamine methyl ester (TMRM) for 10 min before the experiment. Subsequently, NAD(P)H autofluorescence (*λ*_Ex_ = 340 nm, *λ*_Em_ = 450 nm) and TMRM fluorescence (*λ*_Ex_ = 560 nm, *λ*_Em_ = 600 nm) were determined with the same concentrations of substrates and effectors described above.

For both H_2_O_2_ emission and concomitant assessment of NAD(P)H and Δ*Ψ*_m_, fluorescence or absorption was measured in the fluorescence plate reader Infinite M200Pro (Tecan, Männedorf, Switzerland) using Falcon^®^ 96-well plate black/clear (Corning Inc., Corning, NY, USA) for Amplex^®^ UltraRed or UV flat bottom Microtiter^®^ plates (Thermo Fisher Scientific, Waltham, Massachusetts, USA) for NAD(P)H and TMRM. We used 60 µg mitochondria per well in triplicate in a total volume of 200 µl respiration buffer at 37 °C.

### Enzyme activities

All enzyme activities were determined by measuring absorption changes of NAD(P)H every minute at 340 nm in a total volume of 200 μL reaction buffer at 37 °C after one freeze/thaw cycle to break mitochondria. For isocitrate dehydrogenase type 2 (IDH2) activity, 5 µg of mitochondria or cytosol was supplemented with (in mM) TRIS–HCl (pH 8.0) 10, NADP^+^ 0.2, MgCl_2_ 5, isocitrate 2. For aconitase activity, 25 µg of mitochondria or cytosol was supplemented with (in mM) TRIS–HCl (pH 7.4) 36, cysteine 0.8, MgCl_2_ 0.4, NADP^+^ 0.2, citrate 30; dependence on H_2_O_2_ was tested by adding H_2_O_2_ to the reaction buffer. For α-KGDH, 10 µg of mitochondria was supplemented with (in mM) KH_2_PO_4_ 25, EGTA 0.5, MgCl_2_ 5, NAD^+^ 1, α-KG 2.5, thiamine pyrophosphate 0.2, rotenone 0.008. The reaction was started by adding 0.1 mM coenzyme A. For malate dehydrogenase (MDH) activity, 10 µg of mitochondria or cytosol was supplemented with (in mM) TRIS–HCl (pH 7.4) 50, NADP^+^ 0.2, MgCl_2_ 5, malate 5.

### Statistical analysis

All values are displayed as means ± standard error of the mean. One-way/two-way ANOVA followed by Bonferroni’s multiple comparisons test and unpaired Student’s t-test were performed using GraphPad Prism version 7.00 for Windows (GraphPad Software, La Jolla, CA, USA).

## Results

### Functional NNT is required for α-KGDH-dependent regeneration of mitochondrial antioxidative capacity

The Krebs cycle enzyme α-KGDH is a major source of NADH within mitochondria, but its contribution to NADPH regeneration and thereby, the maintenance of mitochondrial antioxidative capacity is not known. To address this, we determined H_2_O_2_ emission from cardiac mitochondria isolated from BL/6N mice in the presence of pyruvate/malate (P/M) or α-KG. The rate of H_2_O_2_ emission did not differ when mitochondria were supplied with P/M or α-KG (Fig. 1a, blue traces; direct comparison in **Supplementary Fig. 1a**). Intriguingly, complex I inhibition with rotenone induced a marked increase in H_2_O_2_ emission from mitochondria supplied with P/M, while it had no effect in the presence of α-KG (Fig. 1a, red traces). Furthermore, the addition of the α-KGDH inhibitor 3-methyl-2-oxovaleric acid (KMV) increased H_2_O_2_ emission from mitochondria supplied with P/M, but not with α-KG, suggesting that the α-KGDH complex is not a major source of ROS under these conditions (Fig. 1a, green traces), but rather contributes to H_2_O_2_ elimination.

Rotenone induces ROS production by blocking the coenzyme Q-binding site of complex I, thereby leading to a back-up of NADH-derived electrons onto the flavin group of the complex, where superoxide is produced by the reaction of electrons with oxygen [24]. To explain why rotenone does not exacerbate H_2_O_2_ emission from mitochondria supplied with α-KG, we hypothesized that NADH derived from the α-KGDH reaction is preferentially used for NADPH regeneration via the NNT, whereas NADH generated by pyruvate and malate dehydrogenase is shuttled to complex I, where it fuels ROS production (scheme in Fig. 1b). To test this hypothesis, we assessed H_2_O_2_ emission under the same conditions in mitochondria isolated from BL/6J mice, a strain carrying a loss-of-function mutation of *Nnt* [37]. In BL/6J mitochondria provided with α-KG as substrate, the rate of H_2_O_2_ emission at baseline and in particular, upon complex I inhibition with rotenone was significantly higher compared to the one observed in the presence of P/M (Fig. 2a, blue and red traces). On the contrary, α-KGDH inhibition with KMV decreased H_2_O_2_ emission, indicating that the α-KGDH complex might become a relevant source of ROS under these conditions (Fig. 2a, green trace). These observations support our hypothesis and indicate that NADPH regeneration becomes exclusively dependent on the NNT reaction when α-KG is provided as substrate (scheme in Fig. 2b).

To further investigate this, we repeated the experiment after incubation of mitochondria with 2,4-dinitrochlorobenzene (DNCB), which leads to alkylation of free glutathione and thereby, depletes mitochondrial antioxidative capacity. As expected, the rate of H_2_O_2_ emission from BL/6N and BL/6J mitochondria treated with DNCB was substantially higher compared to mitochondria with an intact antioxidative capacity (Fig. 3a–c, direct comparison with Fig. 1 shown in **Supplementary Fig. 1**). In the presence of P/M, H_2_O_2_ emission did not differ between BL/6N and BL/6J mitochondria and was exacerbated to the same extent by rotenone addition in both strains (Fig. 3a–c). Intriguingly, when DNCB-treated mitochondria were supplied with α-KG, complex I blockade with rotenone increased H_2_O_2_ emission from BL/6J, but not from BL/6N mitochondria, and the latter strain displayed a rate of H_2_O_2_ emission that was comparable to the one of BL/6J mitochondria with an intact antioxidative capacity (Fig. 3a-c, **Supplementary Fig. 1**).

To explain why DNCB increases H_2_O_2_ emission only in the presence of P/M as substrates, we reasoned that under these conditions, both the isocitrate dehydrogenase (IDH) type 2 (IDH2, i.e. the NADP^+^-dependent isocitrate dehydrogenase, IDH3 being NAD^+^-dependent) and NNT contribute to maintain the antioxidative capacity via NADPH regeneration (Fig. 1b, left panel; **Supplementary Fig. 1**). We previously reported that IDH2 is the main source of NADPH in cardiac mitochondria and its activity does not differ between BL/6N and BL/6J mitochondria [28]. We also did not detect differences in the activity of the other two Krebs cycle enzymes upstream of α-KGDH, i.e. citrate synthase and aconitase (**Supplementary Fig. 2**). In contrast, the observation that DNCB does not affect the rate of H_2_O_2_ emission from BL/6N mitochondria supplied with α-KG corroborates the hypothesis that NADPH regeneration is exclusively dependent on the NNT under these conditions, and that the NADH generated from α-KGDH is shuttled to a higher extent to the NNT than to the ETC (Fig. 1b, right panel).

### Pyruvate/malate and α-ketoglutarate yield different maxima of NAD(P)H, membrane potential and respiration in isolated cardiac mitochondria

The mitochondrial formation of ROS critically depends on the NADH/NAD^+^ ratio and the mitochondrial membrane potential (ΔΨ_m_) [24]. Previous studies investigating ROS production by the α-KGDH complex employed conditions with full reduction of the NADH/NAD^+^ pool, but did not clarify to what extent α-KG by itself is able to reduce NAD(P)^+^ and generate ΔΨ_m_. To interrogate whether the differences in H_2_O_2_ emission from BL/6N and BL/6J mitochondria might be explained by differences in redox state or membrane potential between the two genotypes, we measured changes in NAD(P)H levels (by autofluorescence) and ΔΨ_m_ (by TMRM fluorescence) in BL/6N and BL/6J cardiac mitochondria supplied with either P/M or α-KG. The addition of P/M to isolated mitochondria rapidly increased NAD(P)H autofluorescence and Δ*Ψ*_m_, independent of the genotype, although we noted a trend toward higher NAD(P)H levels in BL/6N compared to BL/6J mitochondria. In the presence of α-KG, the net increase and rate of increase in NAD(P)H and ΔΨ_m_ were substantially lower compared to the ones induced by P/M in both strains (Fig. 4a). In mitochondria supplied with P/M, adenosine diphosphate (ADP) addition partially dissipated ΔΨ_m_ and oxidized the NAD(P)H pool, whereas a mild increase in NAD(P)H levels in response to ADP was observed in BL/6N and BL/6J mitochondria respiring on α-KG (Fig. 4b). This effect might be attributable to the ADP-mediated stimulation of the α-KGDH complex [18].

In a separate experiment, the addition of substrates was followed by complex I inhibition with rotenone, which completely dissipated ΔΨ_m_ independent of the genotype and substrate used (Fig. 4c). In mitochondria supplied with P/M, rotenone did not affect NAD(P)H levels, implying that the NAD(P)H/NAD(P)^+^ pool is almost fully reduced in the presence of P/M. In contrast, we observed a marked increase in NAD(P)H autofluorescence after rotenone addition when α-KG was used as substrate (Fig. 4c). Again, we did not detect differences between BL/6N and BL/6J mitochondria. Finally, we compared the capacity of P/M and α-KG to support mitochondrial respiration. In agreement with the lower efficiency of α-KG-induced NAD(P)H production, ADP-stimulated (i.e., state 3) respiration was substantially lower when mitochondria were respiring on α-KG (Fig. 4d). Mitochondrial O_2_ consumption was similar in BL/6J and BL/6N mitochondria respiring on P/M, but tended to be higher in BL/6J than in BL/6N mitochondria supplied with α-KG alone. Altogether, these results indicate that the pronounced difference in H_2_O_2_ emission between BL/6N and BL/6J mitochondria in the presence of α-KG alone and upon complex I inhibition is neither explained by differences in NADH/NAD^+^ ratio nor in membrane potential. On the contrary, NAD(P)H levels tended to be higher in BL/6N compared to BL/6J mitochondria, which would induce an even larger increase in ROS production by virtue of the higher NADH/NAD^+^ ratio.

### Impaired α-KGDH activity does not affect ROS emission from isolated mitochondria

To further investigate the functional role of the α-KGDH reaction in cardiac mitochondria, we used a mouse model characterized by global heterozygous deletion of the gene encoding the DLST subunit of the α-KGDH complex (*Dlst*^+/−^ mice). While homozygous *Dlst* knockout is embryonically lethal, *Dlst*^+/−^ mice exhibit ~ 25% lower α-KGDH activity in the heart (**Supplementary Fig. 3a**). However, we observed that after cardiac myocyte isolation, the differences in α-KGDH activity between *Dlst*^+/−^ and WT mice, as sampled *from the very same* digested hearts as the cell experiments were performed from, were slightly less pronounced (**Supplementary Fig. 3b**), possibly explained by post-translational modifications of α-KGDH induced by the isolation procedure (i.e., oxidation [35]. Therefore, we re-grouped WT and *Dlst*^+/−^ myocytes into those with low, intermediate, and high α-KGDH activity depending on whether the α-KGDH in the respective tissue was below 60%, between 60 and 80% or above 80%, respectively, compared to the one of WT myocytes with the highest α-KGDH activity (Fig. 5a). The activity of aconitase, IDH2, and malate dehydrogenase did not differ between the three groups (**Supplementary Fig. 3c-e**). Because α-KGDH activity was only partially reduced, α-KG alone supported respiration of cardiac mitochondria isolated from *Dlst*^+/−^ mice, although O_2_ consumption tended to be lower in the low- and intermediate-α-KGDH activity groups compared to mitochondria with high α-KGDH activity (Fig. 5b).

To address the consequences of impaired α-KGDH activity on mitochondrial ROS formation, we measured H_2_O_2_ emission from *Dlst*^+/−^ cardiac mitochondria supplied with α-KG. In the presence of α-KG alone and upon addition of increasing concentrations of ADP, mitochondrial H_2_O_2_ emission did not differ between the three groups of α-KGDH activity (Fig. 5c), thus arguing against α-KGDH being a dominant source of mitochondrial ROS under these conditions.

### Decreased α-KGDH activity oxidizes mitochondrial redox state but does not affect ROS emission in intact cardiac myocytes

The ATP demand of cardiac myocytes changes continuously in response to variations in cardiac workload, and Ca^2+^ plays a key role in the process of energy supply-and-demand matching in the heart [3]. In fact, elevations of cardiac workload (and thus, ATP demand) are accompanied by an increase in the amplitude and frequency of cytosolic Ca^2+^ transients that drive Ca^2+^ accumulation in the mitochondrial matrix, where Ca^2+^ stimulates pyruvate-, isocitrate- and α-KG dehydrogenases to regenerate NAD^+^ to NADH. To interrogate whether decreased α-KGDH activity has an impact on mitochondrial redox state in cardiac myocytes challenged with an increase in energy demand, we measured the autofluorescence of NAD(P)H and oxidized flavin adenine dinucleotide (FAD) in cardiac myocytes harvested from *Dlst*^+/-^ and WT mice during an experimental stress protocol that simulates a physiological transition of cardiac workload. Specifically, cardiac myocytes were superfused with the β-adrenergic receptor agonist isoproterenol, and pacing frequency was increased from 0.5 to 5 Hz for 180 s. As previously observed, the increase in ATP demand accelerates electron flow from mitochondrial pyridine nucleotides to the ETC complexes, thereby inducing a mild oxidation of the NAD(P)H/NAD^+^ and FADH_2_/FAD redox states (Fig. 5d, e). Interestingly, mitochondrial redox state was not different at baseline (0.5 Hz stimulation frequency), but underwent a substantially larger oxidation in cardiac myocytes with low α-KGDH activity compared with the ones with high or intermediate α-KGDH activity in response to this workload transition (Fig. 5d, e). Cardiac myocytes contractility and relaxation kinetics did not differ depending on α-KGDH activity (**Supplementary Fig. 4**).

Because the Ca^2+^-dependent stimulation of Krebs cycle dehydrogenases is also relevant for the regeneration of mitochondrial H_2_O_2_-detoxifying capacity, we investigated whether impaired α-KGDH activity aggravated the overflow of ROS in intact cardiac myocytes. To this end, cells were loaded with the ROS indicator DCF and subjected to the experimental stress protocol described above. Accumulation of DCF fluorescence did not differ between the three groups of α-KGDH activity, indicating similar levels of ROS emission (Fig. 5f). However, when cardiac myocytes were challenged with external H_2_O_2_ to assess their H_2_O_2_-eliminating capacity, DCF fluorescence increased almost twofold in cardiac myocytes with high α-KGDH activity compared to the ones with low and intermediate α-KGDH activity (Fig. 5g). Altogether, these results indicate that decreased α-KGDH activity impairs energetic adaptation to elevated cardiac workload, but does not affect mitochondrial antioxidative capacity under physiological conditions.

## Discussion

Oxidative stress results from an imbalance between ROS formation and their elimination by cellular antioxidative systems. Mitochondria are a major source of ROS in the heart, and a wealth of preclinical evidence indicates that both increased ROS formation and impaired mitochondrial antioxidative capacity are implicated in the progression of heart failure [3]. Within mitochondria, complex I and III of the respiratory chain are generally considered the major sources of ROS, but other potential sites of ROS production have been identified [24]. Specifically, it has been proposed that mitochondrial dehydrogenases, and in particular the α-KGDH complex, might become dominant ROS sources under conditions of high NADH/NAD^+^ ratio [30, 34, 38]. In fact, the flavin groups of complex I and other matrix dehydrogenases become fully reduced under these conditions, thus maximizing the possibility of ROS formation upon leakage of electrons to O_2_. On the other hand, NADPH produced by the Krebs cycle dehydrogenases and the NNT reaction is pivotal to regenerating mitochondrial antioxidative capacity in the heart, and studies evaluating α-KGDH-dependent ROS production were performed in skeletal muscle and brain mitochondria, where NNT activity is negligible compared to the heart [15, 16, 28]. Here, we investigated the role of α-KGDH-derived NADH in the regulation of mitochondrial redox state and ROS emission in isolated cardiac mitochondria and intact cardiac myocytes.

### NADH produced by α-ketoglutarate dehydrogenase is preferentially shuttled to NADPH via the NNT under non-phosphorylating conditions

The central observation of our work is that in cardiac mitochondria from NNT-competent BL/6N mice, H_2_O_2_ emission is equally low with P/M or α-KG as substrates, but complex I inhibition with rotenone exacerbates H_2_O_2_ emission exclusively in mitochondria supplied with P/M, while having no effect on mitochondria supplied with α-KG (Fig. 1a). Conversely, in NNT-deficient BL/6J mitochondria, H_2_O_2_ emission is higher with α-KG than P/M as substrate, and further potentiated by complex I blockade (Fig. 2a). Importantly, addition of rotenone completely dissipated ΔΨ_m_ (Fig. 4c) and thus, the difference in H_2_O_2_ production upon complex I inhibition cannot be accounted for by a difference in mitochondrial membrane potential between the two strains. These results suggest that NADH derived from the α-KGDH reaction is preferentially used for NADPH regeneration via the NNT, which represents the dominant source of NADPH in the presence of α-KG alone. In fact, the observation that rotenone cannot increase H_2_O_2_ emission from BL/6N mitochondria supplied with α-KG (Fig. 1a) implies that the mitochondrial antioxidative capacity is preserved, while ROS formation at the respiratory chain (or in any other mitochondrial ROS sources that can be fueled by NADH) is blunted under these conditions (scheme in Fig. 1b). In contrast, the rotenone-induced increase in H_2_O_2_ emission from BL/6J mitochondria supplied with α-KG is higher than that observed in the presence of P/M (Fig. 2a), indicating that (i) mitochondrial H_2_O_2_-eliminating capacity is depleted and (ii) ROS production is exacerbated by shuttling of α-KGDH-derived NADH toward complex I and, potentially, the flavin groups of mitochondrial dehydrogenases.

This concept is substantiated by our observations in mitochondria depleted of their antioxidative capacity via pre-incubation with DNCB. In the presence of P/M, DNCB treatment led to a substantial increase in rotenone-induced H_2_O_2_ emission from both BL/6N and BL/6J mitochondria compared with mitochondria with an intact antioxidative capacity. This implies that P/M metabolism produces NADPH required for regenerating the reduced form of peroxiredoxin and glutathione reductase and to protect catalase from H_2_O_2_-mediated inactivation independent of the NNT, most likely via the IDH2 reaction. This is in agreement with our previous observation that NADP^+^ sensitivity and maximal efficacy of IDH2 are higher than of NNT in cardiac mitochondria [26]. It must also be noted that aconitase, the Krebs cycle enzyme upstream of IDH, is particularly sensitive to oxidative damage [7]. Therefore, under conditions of oxidative stress, as in the presence of rotenone in our experiments or with a functional block at complex I as reported for heart failure [11], ROS-mediated aconitase inactivation might further limit the flux of substrates into IDH and α-KGDH and thereby, further compromise mitochondrial antioxidative capacity, eliciting an adverse feed-forward mechanisms of mitochondrial oxidative stress.

In contrast, DNCB treatment did not affect the (already high) rate of H_2_O_2_ emission from BL/6J mitochondria supplied with α-KG (Figs. 2a, 3b, directly compared in **Supplementary Fig. 1b**), indicating that α-KG alone does not support NADPH regeneration in the absence of a functional NNT (scheme in Fig. 2b). Consequently, mitochondrial H_2_O_2_-eliminating capacity is extremely low under these conditions and not further depleted by DNCB. Furthermore, also when mitochondrial antioxidative systems were exhausted with DNCB, rotenone did not exacerbate H_2_O_2_ emission from BL/6N mitochondria (Fig. 3a). This further substantiates the notion that NADH produced by α-KGDH shuttles preferentially to the NNT for NADPH formation and not (or to a much lesser extent) to complex I (scheme in Fig. 1b). Otherwise, one would have expected an increase in ROS formation in the face of DNCB-induced depletion of antioxidative capacity. Altogether, these results indicate that the NNT reaction is a dominant source of NADPH when mitochondria are supplied with α-KG alone and, upon complex I inhibition, it functions as an antioxidative “safety valve escape” that concomitantly regenerates mitochondrial H_2_O_2_-eliminating systems and prevents NADH-mediated ROS production by the flavin groups of complex I and mitochondrial dehydrogenases.

The results of a recent study based on a mouse model of homozygous deletion of the *Nnt* (*Nnt*^−/−^ mice) corroborate this concept. In their experiments, Ronchi and colleagues [32] exposed liver mitochondria from WT and *Nnt*^−/−^ mice to an exogenous organic peroxide (*t*-BOOH) to induce a transient oxidation of the NAD(P)H pool. Subsequently, they monitored the time required to restore baseline levels of NAD(P)H in the presence of different substrate couples, including P/M and glutamate/malate (G/M). Glutamate enters mitochondria in exchange for aspartate and is channeled into the Krebs cycle after conversion to α-KG, and thus relies on the α-KGDH for further oxidation to succinyl-CoA. In WT mitochondria, NADPH regeneration was equally efficient independent of the substrate used. In contrast, mitochondria lacking a functional NNT could restore baseline levels of NAD(P)H only when supplied with P/M as substrates, whereas no recovery was observed with G/M [32]. These results indicate that, when mitochondria are supplied with G/M or α-KG alone, NADPH regeneration under non-phosphorylating conditions is largely dependent on the NNT.

### Reduced α-ketoglutarate dehydrogenase activity does not affect ROS elimination in isolated cardiac myocytes

To examine whether α-KGDH-derived NADH regulates mitochondrial redox balance under more physiological conditions, we investigated how α-KGDH activity influences ROS emission and mitochondrial redox state of isolated mitochondria and cardiac myocytes challenged with an increase in energy demand. In cardiac mitochondria respiring on α-KG, lower α-KGDH activity is associated with a trend toward decreased O_2_ consumption (Fig. 4d), but does not affect H_2_O_2_ emission (Fig. 5c). In isolated cardiac myocytes, lower α-KGDH activity does not influence mitochondrial redox state at baseline, but impairs metabolic adaptation to increased workload, as evidenced by the pronounced oxidation of the NAD(P)H/FAD ratio elicited by concomitant β-adrenergic stimulation and elevated pacing frequency (Figs. 5d, e). This observation highlights the importance of the Ca^2+^-stimulated Krebs cycle dehydrogenases for NAD(P)H regeneration during elevated cardiac workload. However, we did not detect an increase in ROS overflow within intact cardiac myocytes with low α-KGDH activity (Fig. 5f), indicating that on the one hand, α-KGDH is unlikely a relevant source of ROS under physiological conditions in cardiac myocytes. On the other hand, these results also suggest that the contribution of α-KGDH-derived NADH and subsequently, NADPH, is not essential for ROS elimination under physiological conditions. This may be explained by the fact that in cardiac myocytes, IDH2 has the highest contribution towards NADPH regeneration compared to NNT and malate dehydrogenase [28].

Together with our observations in isolated mitochondria, this result supports the emerging concept that the contribution of the NNT reaction to mitochondrial antioxidative capacity is higher under non-phosphorylating conditions (e.g., complex I inhibition with rotenone) than during states of high energy demand [32]. Such conditions may apply to pancreatic islet cells, in which glucose-dependent ATP production serves to close ATP-dependent K^+^ channels and thereby, increase Ca^2+^ influx and insulin release. In these cells, ATP demand is lower than in cardiac myocytes, resembling closer non-phosphorylating conditions, and in fact, NNT deletion provokes ROS formation and disruption of glucose-induced insulin release [33, 37].

### Implications for cardiac hypertrophy and failure

Intriguingly, a recent study by our groups indicates that pathological elevations of cardiac afterload lead to a decrease in α-KGDH activity [8]. Mechanistically, mouse models of pressure overload and left ventricular biopsies of patients with aortic stenosis display an increased expression of microRNA-146a, which targets and induces degradation of DLST, the E2 component of the α-KGDH complex. From a pathophysiological standpoint, conditions that downregulate α-KGDH expression and/or activity impair energetic adaptation and NADPH regeneration, which may have an adverse effect on intracellular redox balance and energetics. Accordingly, genetic deletion of microRNA-146a restored α-KGDH activity and partially prevented metabolic and structural remodeling of the pressure-overloaded mouse heart [8]. Our findings in isolated cardiac myocytes suggest that the protective effect obtained by maintaining α-KGDH activity might be mediated by the preservation of mitochondrial redox balance under conditions of elevated cardiac workload.

Heart failure is common, costly, disabling, and deadly [23]. Treatment of heart failure with reduced ejection fraction, the clinical correlate of the animal models used to investigate heart failure, is largely based on medications that reduce the detrimental activation of the renin–angiotensin–aldosterone and sympathetic nervous system [29]. Although experimental evidence supports a crucial role of mitochondrial dysfunction in the progression of heart failure, no specific mitochondria-targeted treatment is currently used in clinical practice. Nevertheless, several clinical trials are currently ongoing to validate preclinical findings in the human setting (e.g. with elamipretide [4, 14]). We believe that our data contribute to shed more light on the complex mitochondrial biology that underlies pathological metabolic remodeling and energy starvation in heart failure.

### Limitations

This study has limitations: first, all experiments were conducted in isolated mitochondria or unloaded cardiac myocytes, thus removing the natural context of the intact, beating heart. In addition, the Amplex^®^ UltraRed assay used in Figs. 1,2,3 reports exclusively extramitochondrial H_2_O_2_, whereas we did not assess ROS *formation* selectively in the mitochondrial matrix. Furthermore, NAD(P)H and ΔΨ_m_ measurements shown in Fig. 4a-c are semiquantitative, and therefore we cannot draw conclusions on the absolute values of NAD(P)H and ΔΨ_m_ in our mitochondria preparation. *Dlst*^+/−^ cardiac myocytes displayed only a modest decrease in α-KGDH activity, and this led us to re-group WT and *Dlst*^+/−^ mice used for our cellular experiments based on the actual activity of the enzyme, rather than their genotype. We acknowledge that an approach based on genetic silencing to reduce the enzymatic activity of α-KGDH would have been preferable; however, because α-KGDH activity was determined in the same digested hearts as the cell experiments were performed from, we believe that the results shown in Fig. 5d-g actually capture the effect of varying degrees of α-KGDH activity on mitochondrial NAD(P)H/FAD redox state and cellular ROS production. Finally, the biological significance of the NAD(P)H/FAD oxidation in the cellular experiments of Fig. 5d and e is presently unclear. However, in previous work, a similar oxidation of NAD(P)H at elevated cytosolic Na^+^ concentrations [22] proved to be of biological significance since preventing such Na^+^-dependent mitochondrial oxidation with an inhibitor of the mitochondrial Na^+^/Ca^2+^ exchanger prevented maladaptive cardiac remodeling, dysfunction, and arrhythmias [19, 20].

## Conclusion

In conclusion, our results indicate that in cardiac mitochondria, the α-KGDH is a sink rather than a source of ROS, since NADH produced from α-KGDH is preferentially shuttled towards the NNT rather than to complex I. Of note, the experiments that identified the α-KGDH complex as a major source of ROS were performed in *skeletal* muscle and *brain* mitochondria. We showed previously that in contrast to *cardiac* mitochondria, these have negligible NNT activity [28], thereby preventing the antioxidative “safety valve escape” of NADH shuttling to NADPH under reducing conditions. Therefore, our data resolve a seeming paradox of α-KGDH being a source or a sink for ROS in mitochondria under various conditions. Furthermore, downregulation of α-KGDH by microRNA-146a during cardiac hypertrophy and failure [5] may contribute to redox imbalance during situations of elevated cardiac workload.

## Electronic supplementary material

Below is the link to the electronic supplementary material.

Supplementary file1 (PPTX 5542 kb)

## Abbreviations

ADP: Adenosine diphosphate
ATP: Adenosine triphosphate
α-KG: α-Ketoglutarate
α-KGDH: α-Ketoglutarate dehydrogenase
CI, CIII and CIV: Complex I, III and IV of the electron transport chain
DCF: 5-(-6)-Chloromethyl-2,7-dichlorohydrofluorescein di-acetate
DLD: Dihydrolipoyl dehydrogenase
DLST: Dihydrolipoyl succinyltransferase
DNCB: 2,4-Dinitrochlorobenzene
ΔΨ_m_: Mitochondrial membrane potential
EC coupling: Excitation–contraction coupling
ETC: Electron transport chain
IDH2: Isocitrate dehydrogenase type 2 (NADP^+^-dependent, mitochondrial)
IDH3: Isocitrate dehydrogenase type 3 (NAD^+^-dependent, mitochondrial
KMV: 3-Methyl-2-oxovaleric acid
Mn-SOD: Manganese-dependent superoxide dismutase
NNT: Mitochondrial nicotinamide nucleotide transhydrogenase
OAA: Oxaloacetate
P/M: Pyruvate/malate
ROS: Reactive oxygen species
TMRM: Tetramethylrhodamine methyl ester
