# Supplementary material for: Selective NADH communication from α-ketoglutarate dehydrogenase to mitochondrial transhydrogenase prevents reactive oxygen species formation under reducing conditions in the heart
Source: Basic Res Cardiol. 2020 Aug 3;115(5):53. doi: 10.1007/s00395-020-0815-1 (PMC7399685; doi:10.1007/s00395-020-0815-1)
Supplement: Supplementary file 1 — Supplementary file1 (PPTX 5542 kb) [file 395_2020_815_MOESM1_ESM.pptx]

## Slide 1
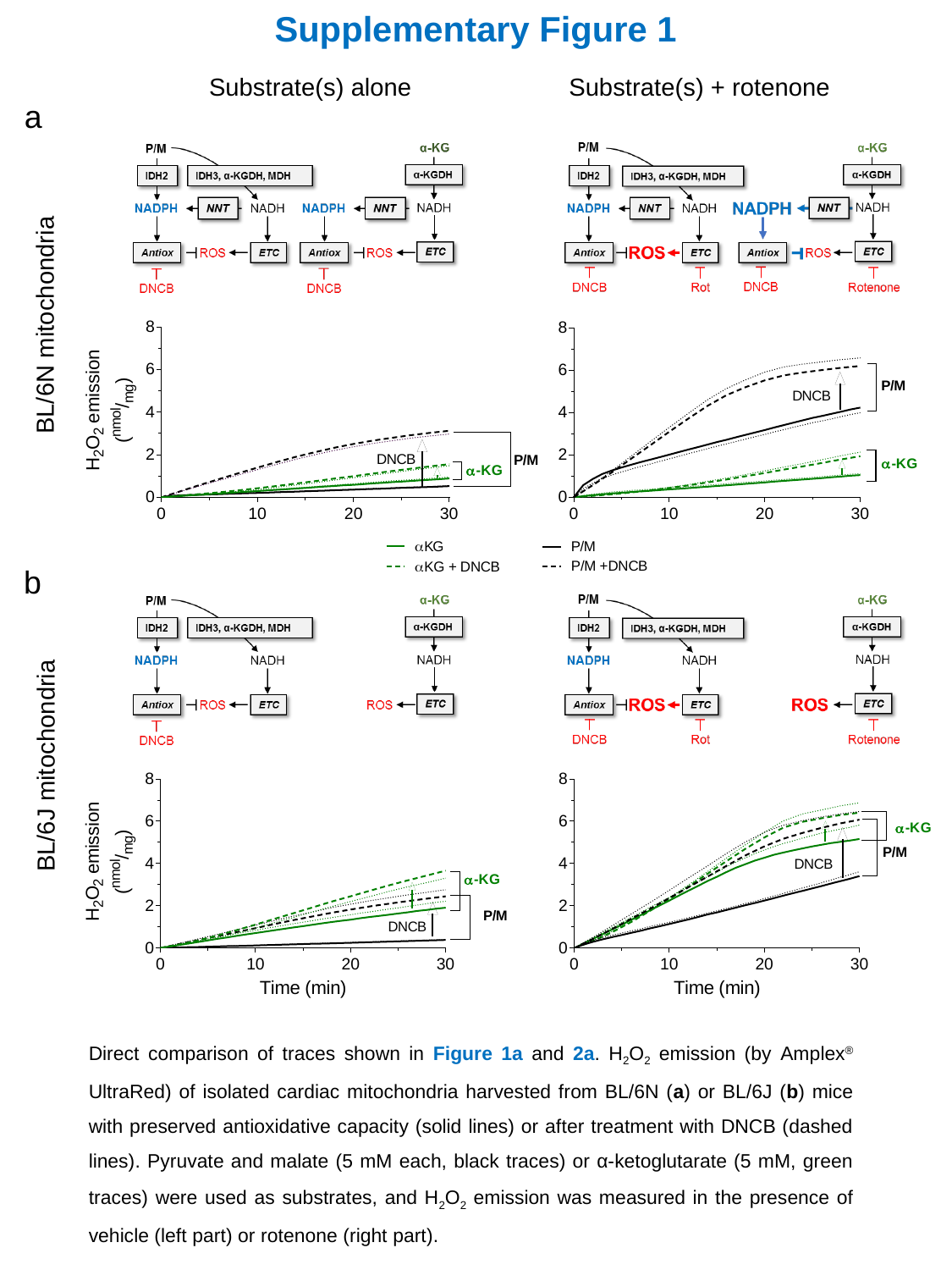

Supplementary Figure 1
Substrate(s) + rotenone
Substrate(s) alone
a
BL/6N mitochondria
b
BL/6J mitochondria
Direct comparison of traces shown in Figure 1a and 2a. H2O2 emission (by Amplex® UltraRed) of isolated cardiac mitochondria harvested from BL/6N (a) or BL/6J (b) mice with preserved antioxidative capacity (solid lines) or after treatment with DNCB (dashed lines). Pyruvate and malate (5 mM each, black traces) or α-ketoglutarate (5 mM, green traces) were used as substrates, and H2O2 emission was measured in the presence of vehicle (left part) or rotenone (right part).

## Slide 2
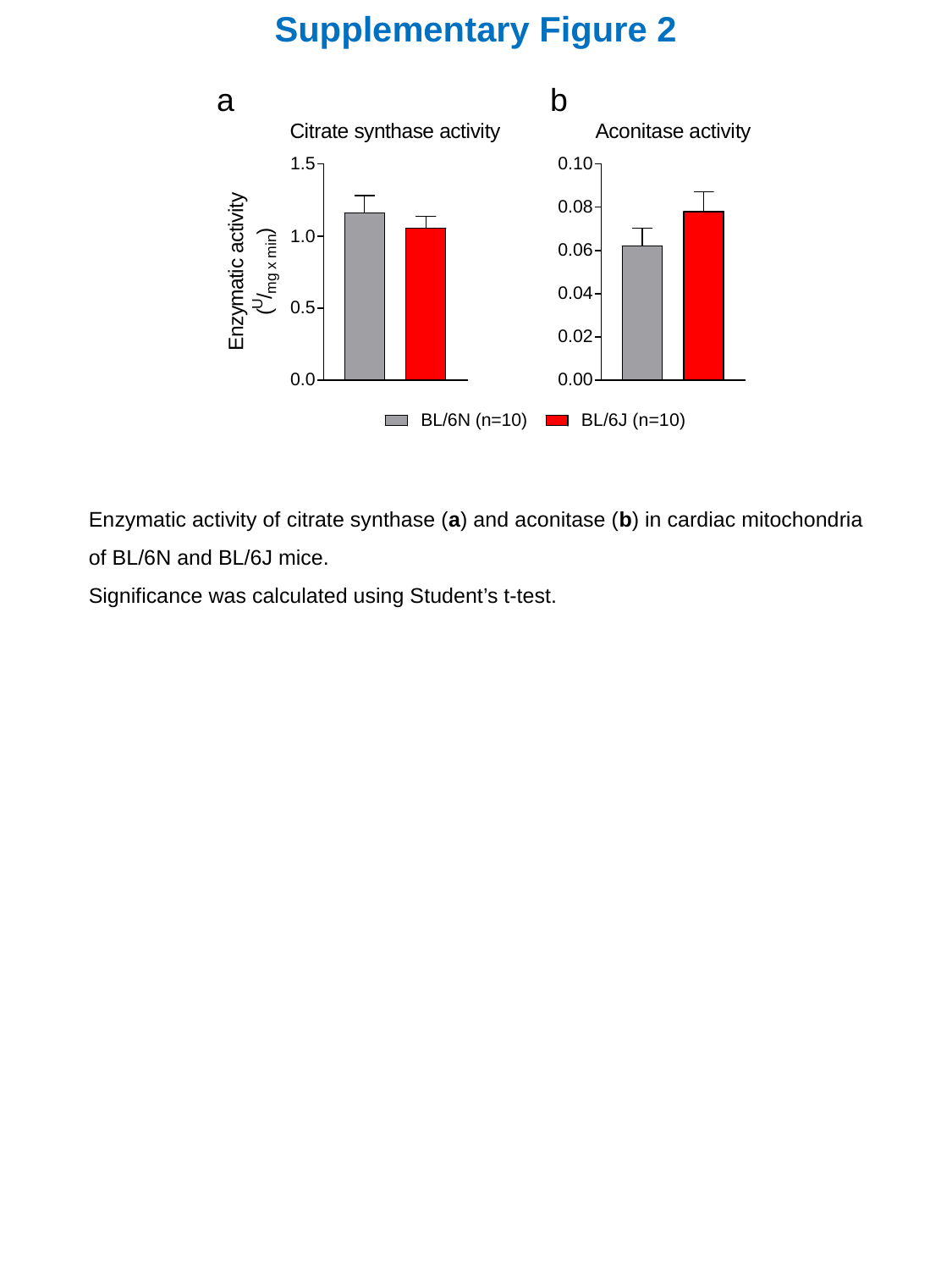

Supplementary Figure 2
a
b
Enzymatic activity of citrate synthase (a) and aconitase (b) in cardiac mitochondria of BL/6N and BL/6J mice.
Significance was calculated using Student’s t-test.

## Slide 3
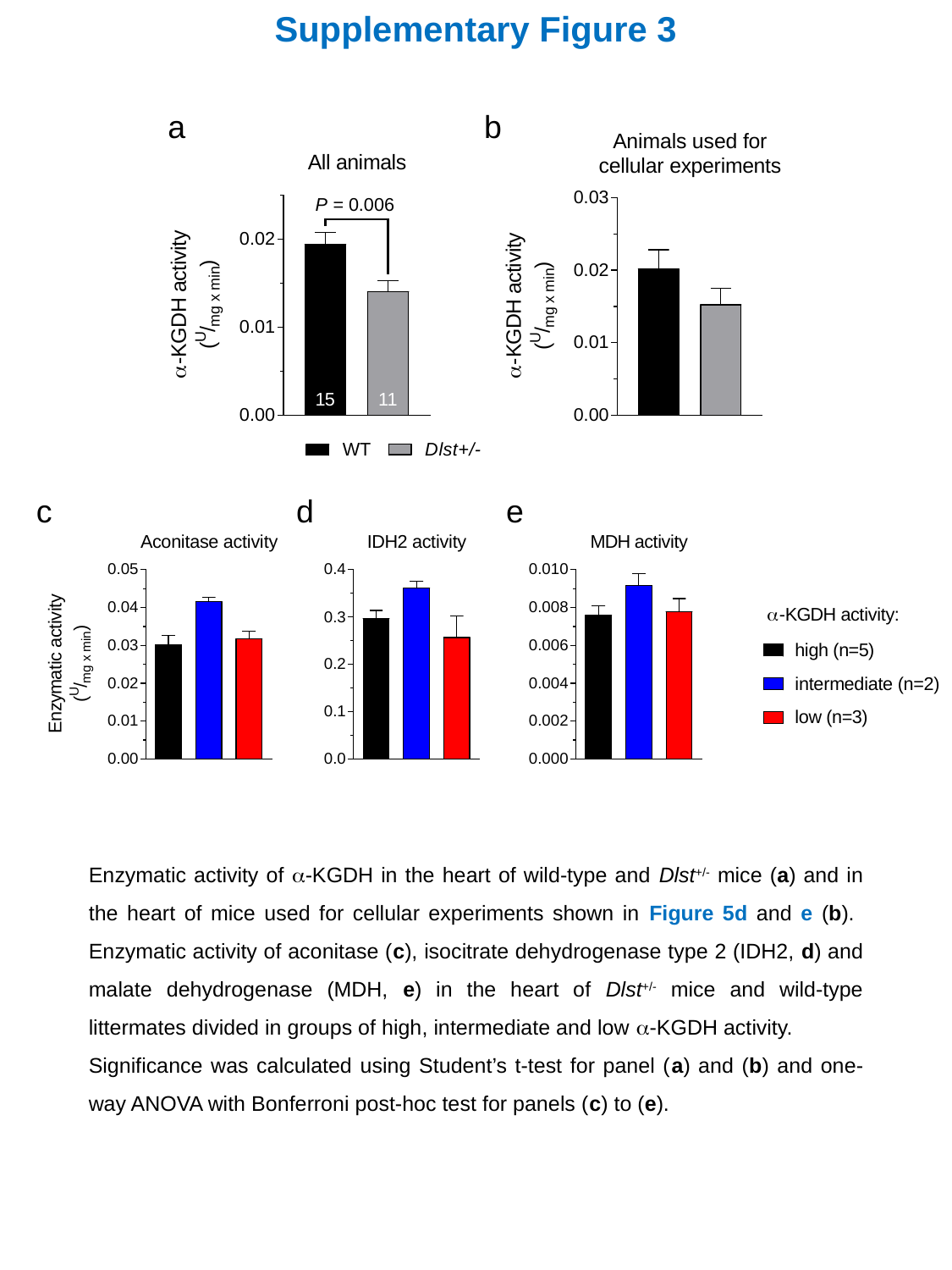

Supplementary Figure 3
a
b
c
d
e
Enzymatic activity of α-KGDH in the heart of wild-type and Dlst+/- mice (a) and in the heart of mice used for cellular experiments shown in Figure 5d and e (b). Enzymatic activity of aconitase (c), isocitrate dehydrogenase type 2 (IDH2, d) and malate dehydrogenase (MDH, e) in the heart of Dlst+/- mice and wild-type littermates divided in groups of high, intermediate and low α-KGDH activity.
Significance was calculated using Student’s t-test for panel (a) and (b) and one-way ANOVA with Bonferroni post-hoc test for panels (c) to (e).

## Slide 4
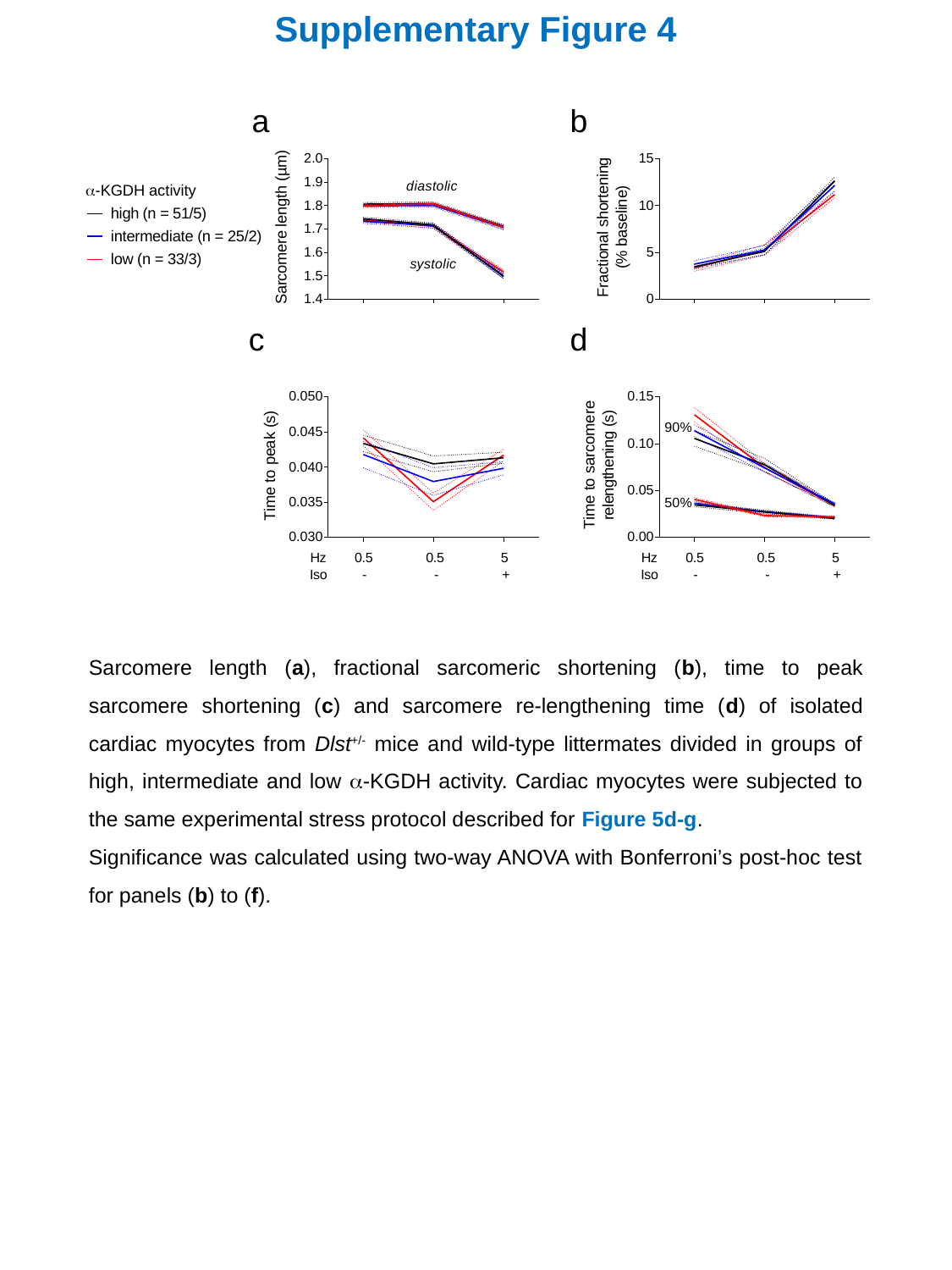

Supplementary Figure 4
a
b
c
d
Sarcomere length (a), fractional sarcomeric shortening (b), time to peak sarcomere shortening (c) and sarcomere re-lengthening time (d) of isolated cardiac myocytes from Dlst+/- mice and wild-type littermates divided in groups of high, intermediate and low α-KGDH activity. Cardiac myocytes were subjected to the same experimental stress protocol described for Figure 5d-g.
Significance was calculated using two-way ANOVA with Bonferroni’s post-hoc test for panels (b) to (f).
